# The impact of social complexity on the visual exploration of others' actions in preschoolers with autism spectrum disorder

**DOI:** 10.1186/s40359-021-00553-2

**Published:** 2021-03-31

**Authors:** F. Robain, N. Kojovic, S. Solazzo, B. Glaser, M. Franchini, M. Schaer

**Affiliations:** 1 Department of Psychiatry, University of Geneva, Faculty of Medicine, Geneva, Switzerland; 2 Fondation Pôle Autisme, Unité de Recherche, 4 place du Cirque, 1204 Geneva, Switzerland

**Keywords:** Eye-tracking, Socio-communicative skills, Social complexity, Parallel Interactive play

## Abstract

**Background:**

Typical development of socio-communicative skills relies on keen observation of others. It thus follows that decreased social attention negatively impacts the subsequent development of socio-communicative abilities in children with autism spectrum disorders (ASD). In addition, studies indicate that social attention is modulated by context and that greater social difficulties are observed in more socially demanding situations. Our study aims to investigate the effect of social complexity on visual exploration of others’ actions in preschoolers.

**Methods:**

To investigate the impact of social complexity, we used an eye-tracking paradigm with 26 typically developing preschoolers (TD, age = 3.60 ± 1.55) and 37 preschoolers with ASD (age = 3.55 ± 1.21). Participants were shown videos of two children engaging in socially simple play (parallel) versus socially complex play (interactive). We subsequently quantified the time spent and fixation duration on faces, objects, bodies, as well as the background and the number of spontaneous gaze shifts between socially relevant areas of interest.

**Results:**

In the ASD group, we observed decreased time spent on faces. Social complexity (interactive play) elicited changes in visual exploration patterns in both groups. From the parallel to the interactive condition, we observed a shift towards socially relevant parts of the scene, a decrease in fixation duration, as well as an increase in spontaneous gaze shifts between faces and objects though there were fewer in the ASD group.

**Limitations:**

Our results need to be interpreted cautiously due to relatively small sample sizes and may be relevant to male preschoolers, given our male-only sample and reported phenotypic differences between males and females.

**Conclusion:**

Our results suggest that similar to TD children, though to a lesser extent, visual exploration patterns in ASD are modulated by context. Children with ASD that were less sensitive to context modulation showed decreased socio-communicative skills or higher levels of symptoms. Our findings support using naturalistic designs to capture socio-communicative deficits in ASD.

## Background

Autism spectrum disorder (ASD) is a group of pervasive neurodevelopmental disorders characterized by impairments in communication, social interactions and the presence of restricted and repetitive behaviors (DSM-5) [1]. A core symptom of ASD, often reported early on by parents, is a difficulty modulating eye contact. Based on this observation, studies have focused on the possible link between atypical visual exploration and social difficulties in autism using eye-tracking technology. The majority of these studies have shown decreased attention to social stimuli in individuals with ASD (often referred to as a lack of social orienting) and less time spent on the eye region compared to people with typical development [2–9] which were more evident when using dynamic (as opposed to static) stimuli [10] and naturalistic social interactions [2]. In addition, studies of high-risk children indicate that atypical social orienting emerges early in development, [8, 11–13] and shapes future developmental trajectories of children with ASD [5, 14, 15].

Decreased social attention among people with ASD compromises their ability to monitor others’ actions early in development [16]. Monitoring others’ actions is critical to social skills that emerge during infancy as watching others actions will give children the opportunity to learn from it and give them the opportunity to develop higher cognitive skills like joint attention, a core impairment in ASD (e.g. [17, 18]). Joint attention is, in turn, key to the subsequent development of other socio-communicative skills [19, 20], which will also support action monitoring by increasing the significance of gaze following, making it central to understanding the mechanisms underlying social development in ASD (for a review of the literature see [21]). Most studies investigating joint attention skills in ASD induce instances of joint attention to quantify the number of times participants accurately follow cues. While such study designs allow for the quantification of joint attention impairments at differential stages in ASD [22–24], they can be unrepresentative of daily life.

Unlike studies using controlled paradigms, Shic et al. [16] used a naturalistic eye-tracking task to track how toddlers monitor others’ activity. They showed participants a video of an adult and a child solving a puzzle together. Twenty-month-old toddlers with ASD looked less at the shared activity and were more often distracted by the background compared to their typically developing (TD) peers. The authors concluded that this decreased monitoring of others' actions can be attributed to decreased joint attention skills in ASD children and “*a limited appreciation for the significance of the shared focus of others*” [16 pp. 5–6]. The use of such a task, one that is closer to everyday life, undoubtedly makes it possible to capture skills that are used in everyday life and to more accurately report the difficulties encountered on a daily basis by children with ASD. The task used by Shic et al. [16] therefore represents an ecologically valid design to investigate others' actions monitoring and subsequent joint attention behaviors in children with ASD. Shic et al. [16] however did not present different social context to investigate how visual exploration is modulated by social complexity.

Social context indeed appears to be a key determinant in divergent visual exploration between children with ASD and TD [25–28]. Studies indicate reduced attention to faces during dyadic bid [26, 28], tickles [28] and joint attention conditions [26],whereas individuals with ASD demonstrate TD-like visual exploration while watching someone make a sandwich [26], play peek-a-boo or sing a song [28]. To further refine diagnostic techniques and shape targeted interventions, it will be essential to further our understanding of the social contexts that accompany atypical visual exploration in ASD. In the current study, we investigate how children monitor others’ actions during passive viewing, one of the primary building blocks of social learning during early development.

Harrop et al. [27] recently used a paradigm inspired from Chevallier et al. [2] that brings together dynamic stimuli with high ecological validity while manipulating context, or what they labelled « *social richness*». The authors presented videos of siblings practicing parallel play or interactive play to observe gender differences during social attention in ASD. In both conditions, males with ASD showed decreased attention to faces, whereas females with ASD demonstrated decreased attention during the interactive condition only and TD-like attention during the parallel condition, thereby reinforcing the idea of context-dependent attentional difficulty in ASD. While similar to Shic et al.’s [16] design, the aim of Harrop et al.'s study was to reveal gender differences during social attention. However, their design would be equally effective for investigating action monitoring and joint attention in children with ASD, due to the combination of ecological social interactions with differential social complexity. Parish-Morris et al. [29] recently reused this design with adults and showed that looking at interactive play compared to parallel play, increased attention to faces in both TD and ASD, though to a lesser extent in the ASD group. However, there are no studies focusing on preschoolers during the critical period for the development of socio-communicative skills. In addition, recent studies highlight the development of several compensatory mechanisms [30, 31] during the development of individuals with autism, making it difficult to assert that the skills observed in adulthood are the same as in childhood. Therefore, how children with ASD visually monitor others’ actions and the way in which this exploration is modulated by social complexity remains unanswered and needs to be investigated considering that many socio-communicative skills emerge during infancy.

In the present study, we aim to combine elements from those previous studies [16, 27, 29] to identify potential differences in the visual exploration of others’ actions in preschoolers with ASD through the manipulation of context. Social complexity, or context, is manipulated in our design by showing videos of two children engaging in either parallel or interactive play. We then compare the time participants spent looking at different areas of the scene (faces, bodies, and objects) and the duration of their fixations, thought to reflect attentional engagement [32], by condition. To assess how participants dynamically view others' actions, we quantify the number of spontaneous gaze shifts between areas of interest (AOI). We hypothesize that children with ASD will exhibit decreased attention to faces in both conditions. However, based on previous results, we expect that the presence of an interaction will increase attention to socially relevant parts of the scene and make visual exploration more dynamic by reducing fixation duration and increasing the number of gaze shifts. Given that numerous studies have demonstrated strong correlations between symptom severity, level of adaptive behavior or cognitive skills and visual exploration patterns in children with ASD [13, 14, 16, 22, 27, 33–35], we also explore the relationship between these clinical measures and visual exploration patterns. We hypothesize that children exhibiting more symptomatology, having lower cognitive skills and lower adaptive scores will attend less to social areas and present less dynamic visual exploration, otherwise known as “*sticky attention*” [36, 37], to non-social areas.

## Method

### Sample

The initial total of acquired recordings for this task was 177. However, to ensure reasonable quality of data we decided to only include recordings where participants attended to both scenes for at least 50% of their total duration, leading to the exclusion of 74 recordings. The exclusion of these recordings led to an imbalanced sex-ratio between our groups including 38 TD females and only 2 females with ASD. Given previous results from Harrop et al. [27] showing sex differences in a similar task and the fact that we did not have enough female participants available for an equally sized sample, we only included males here. Our final sample included 63 preschool-aged males split in two age-matched group of 26 TD children aged 3.60 years (SD = 1.55) and 37 children with ASD aged 3.55 years (SD = 1.21, see Table 1). All children were included in the longitudinal Geneva Autism Cohort described in previous publications [14, 38–40]. Participants with ASD had received a clinical diagnosis of ASD according to the DSM-5 [1] before their inclusion in the study. In addition, all participants were assessed using the Autism Diagnostic Observation Schedule-G, or 2nd edition (ADOS) [41, 42] to re-confirm their diagnoses using a standardized tool. Participants’ parents provided written consent before the start of the evaluations in accordance with protocols approved by the institutional review board of the University of Geneva.

**Table 1:** Sample demographics

N = 63 (♂)	TD (n = 26)	ASD (n = 37)	*p*
Age	3.60 ± 1.55	3.55 ± 1.21	.888
ADOS			
RRB^a^	2.36 ± 2.06	8.08 ± 1.57	< .001
SA	1.16 ± 0.72	6.16 ± 2.02	< .001
Total	1.12 ± 0.44	6.81 ± 2.04	< .001
VABS-II			
Communication skills	103.96 ± 10.97	75.92 ± 14.90	< .001
Socialization skills	103.92 ± 10.49	75.76 ± 10.09	< .001
Daily living skills	104.08 ± 8.22	75.95 ± 10.68	
Motor skills	104.33 ± 12.58	83.84 ± 11.41	< .001
Best estimate IQ^b^	99.49 ± 11.04	62.94 ± 20.39	< .001

^a^RRB severity score scale goes from 1 to 10 but doesn’t include intermediate scores (2–3–4)

^b^Best Estimate IQ was obtained from either the Psycho-Educational Profile, third edition (PEP-3; [46], Mullen Scales of Early Learning (MSEL) [47] or the Wechsler Preschool and Primary Scale of Intelligence, fourth edition (WPPSI-IV) [48] see Method section

### Procedure and clinical measures

We applied the calibrated scores algorithm to our ADOS evaluation [43, 44] to quantify Restricted and Repetitive Behaviors (RRB), Social Affect (SA) and total symptom severity. These calibrated severity scores allow for the comparison of children with various developmental, language and cognitive levels regarding symptom severity and for a comparison between ADOS versions. All ADOS were administered by a trained examiner and scored with a qualified research reliable ADOS examiner. To assess adaptive functioning, examiners completed the Vineland Adaptive Behavior Scales, second edition (VABS-II) [45] with participants’ parents. To assess cognition, children were assessed using the Psycho-Educational Profile, third edition (PEP-3) [46], Mullen Scales of Early Learning (MSEL) [47] or the Wechsler Preschool and Primary Scale of Intelligence, fourth edition (WPPSI-IV) [48] according to developmental level, chronological age and language skills. We subsequently used an approach described by Howlin et al. [49] and Kojovic et al. [38] to obtain a Best Estimate Intellectual Quotient (BEIQ) as an estimation of participants’ cognitive skills at time of visit.

### Eye-tracking task and measures

**Fig. 1:**
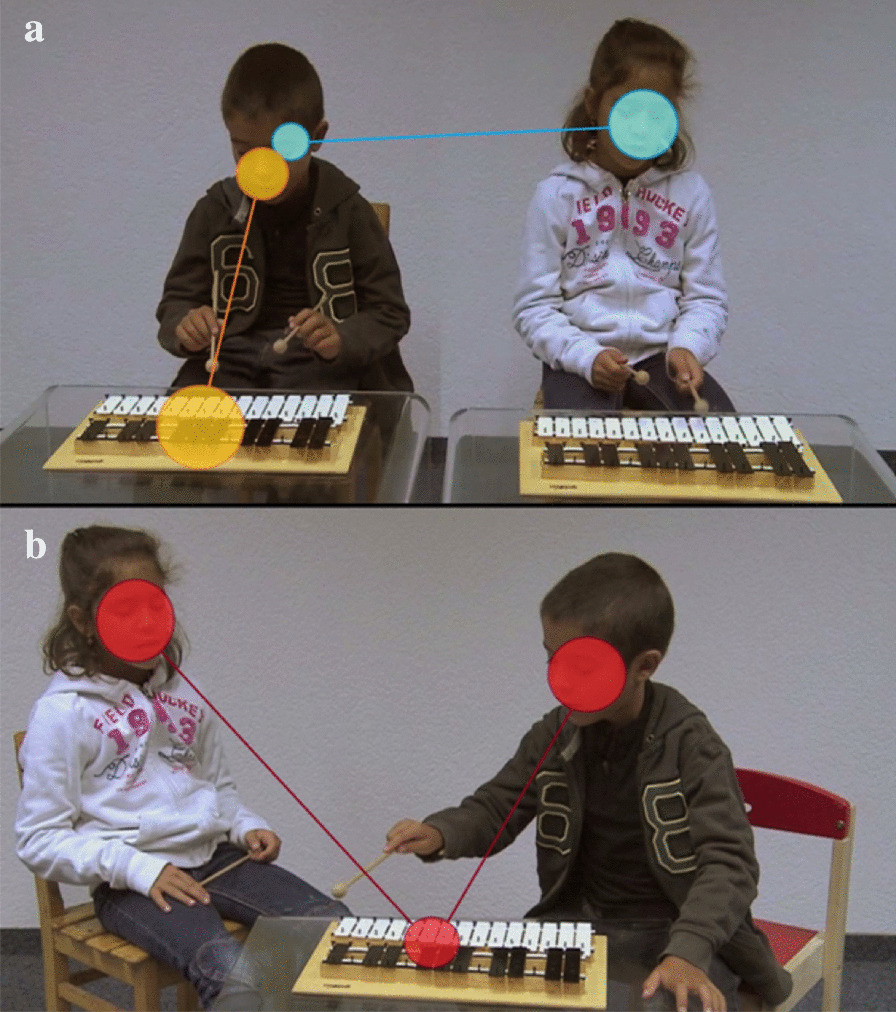
Eye-tracking stimuli. Dots and lines reproduce recorded gaze data. Dots represent fixations with size of the dots proportional to fixation duration. Lines between dots represent saccades. **a** Parallel play scene: orange dots reproduce F-O shift and blue dots reproduce F-F shift; **b** Interactive play scene: red dots reproduce F-O-F shift

Our eye-tracking task included two conditions:

- *the Parallel condition* (see Fig. 1a) showed two children playing independently on a xylophone for 56 s. The two children who figured in the video were told to play as they wished and to look at the xylophone throughout the video. They were filmed separately to avoid subtle interactions and non-verbal communication passing between them. Videos were later edited into a film where they figured playing side by side.
- *the Interactive condition* (see Fig. 1b) showed the same two children taking turns on one xylophone together for a period of 60 s. Once again, they received no prior instructions before the filming, and they played freely together. During this condition, they sometimes looked at each other to establish turn-taking.

Children in the scenes were seven years old twins, a boy and a girl. We chose siblings because we wanted them to be used to playing together, so that their interactions would be as naturalistic as possible. Both scenes took place on a neutral white background to avoid distractions, and used the same material (table, mallets, xylophone). As children were playing the instrument, both scenes included musical tones but no verbal communication at all.

The task was administered using Tobii Studio software 3.1.6 on a TX300 eye-tracker which allows high-rate sampling (300 Hz) on a 1920 × 1200 pixel screen. Participants sat at approximately 60 cm from the screen, alone when possible or on a parent’s lap if needed. After completing a five-point calibration designed specifically for young participants, they looked freely at the screen without any instructions. Fixations and saccades (see [50] for a detailed description) were later defined using the Tobii IV-T Fixation filter [51], which categorizes saccades and fixations using a velocity threshold of 30°/sec. Fixations that were shorter than 60 ms were discarded and adjacent fixations within 75 ms and a maximum of 0.5° were merged. An average of the right and left eyes was used to define fixations and the velocity calculator was set to 20 ms. Dynamic AOI were drawn on faces, bodies, objects (mallets and xylophone) and around the entire scene. From these AOI, we extracted and investigated several parameters, including percentage of fixations (the percentage of time spent in an AOI corrected for the total fixation time on a given scene), fixation duration (averaged using medians in order to reduce skewness while remaining representative of the distribution) and the number of gaze shifts. For the number of gaze shifts, we defined three potential types of gaze shifts between AOI: Face to Face shifts (F-F, see Fig. 1a) for quantifying participants’ attempts at following non-verbal communication cues, Face to Objects shifts (F-O, see Fig. 1a) for quantifying joint attention behaviors, and Face to Objects to Face shifts (F-O-F, see Fig. 1b) reflecting three-step joint attention gaze shifting. The number of gaze shifts were established using custom MATLAB v. 2018b scripts and data extracted from Tobii replays. Interrupted gaze (e.g., fixations on the background during shifts from one face to the other) or data that was lost when shifting from one AOI to another were discarded. Only complete data representing a fixation followed by a saccade and then a final fixation on the AOI as described above were included.

### Analysis strategy

Apart from the number of gaze shifts between AOI, which were calculated using custom MATLAB scripts, all analyses were conducted using IBM SPSS Statistics for MacIntosh, Version 24.0 (Armonk, NY:IBM Corp.) and graphs were plotted using Graphpad Prism 8.0 (GraphPad Software, La Jolla California USA, www.graphpad.com). To ensure that observed differences were not due to a difference in total time spent watching a scene between the groups, we first verified that both groups attended the scene for equal amounts of time. We investigated the effects of Condition (Parallel vs Interactive) and Diagnosis (ASD vs TD), as well as interactions between them in a 2 (Condition) × 2 (Diagnosis) design. We used repeated measures linear general model when investigating at percentages of time spent on AOI and fixation durations. When investigating the number of gaze shifts between AOI, we used a subsample of children, excluding children who did not look at least once at faces during both conditions (*n* = 17; ASD = 14, TD = 3) in order to specifically investigate how children with ASD, who generally demonstrate interest in faces, dynamically monitor others’ actions. To investigate the effect of Condition and Diagnosis on gaze shifting, we performed generalized estimation equations with a Poisson distribution model. Finally, we performed Spearman correlations between clinical measures (ADOS severity scores, VABS-II scores and BEIQ) and eye-tracking measures to investigate potential relationships between ASD phenotype, adaptive functioning, cognitive skills and visual exploration patterns in the ASD group. When investigating correlations between the number of gaze shifts and clinical measures, we used the subsample of ASD children described above for the same reasons.

## Results

Our analyses revealed that the ASD and TD groups attended to both scenes for equivalent amounts of time, we did not identify an effect of diagnosis (F(1,61) = 3.496; *p* > 0.05) on the percentage of time spent looking at the scenes. However, we identified a main effect of condition (F(1,61) = 4.307; *p* = 0.042; ηp^2^ = 0.066), children attended to the interactive condition (mean = 81.918, SE = 1.301) slightly more than the parallel condition (mean = 78.248, SE = 1.548). Finally, we did not detect an interaction between diagnosis and condition (F(1,61) = 1.071; *p* > 0.05).

### Fixation percentage

**Fig. 2:**
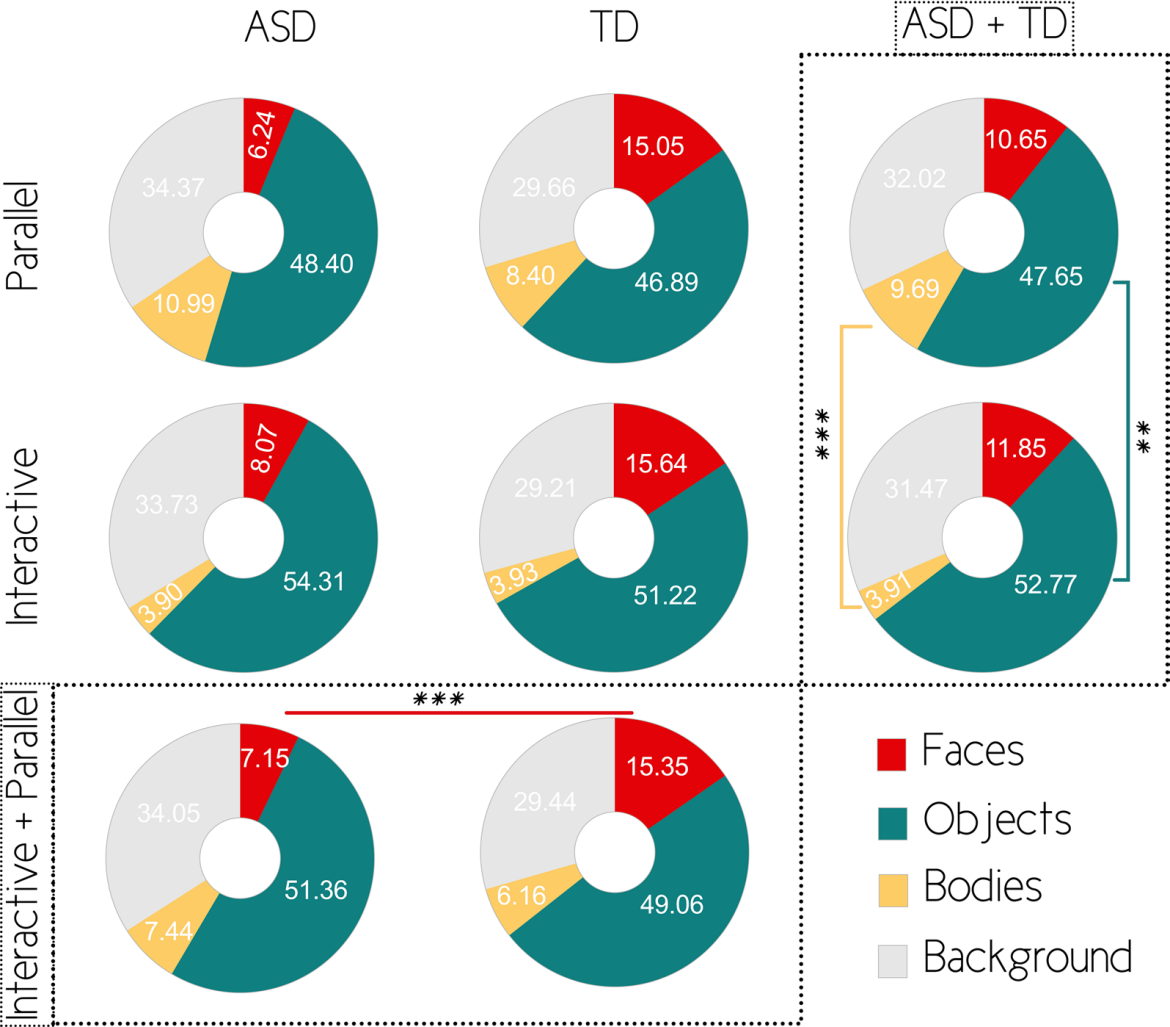
Percentage of time spent per AOI as a function of condition (in lines) and group (in columns). Brackets represent significant differences *p < .05; **p < .01; ***p < .001

Faces (see Table 2 and Fig. 2): We identified an effect of diagnosis on the time spent looking at faces (F(1, 61) = 21.686; *p* < 0.001, ηp^2^ = 0.262), with ASD children (mean = 7.138, SE = 1.135) looking less at faces compared to TD children (mean = 15.364, SE = 1.135). We did not identify any effect of condition (F(1,61) = 1.431; *p* > 0.05) nor an interaction between diagnosis and condition (F(1,61) = 0.384; *p* > 0.05).

**Table 2:** Repeated measures linear general model on fixation percentage

Measure	Source	*df*	MS	F	*p*	ηp^2^	Observed power
Percentage on faces							
Within-subject effects	Condition	1	44.460	1.431	.236	.023	.218
	Condition × Diagnosis	1	11.925	.384	.538	.006	.094
	Error	61	31.065				
Between-subject effects	Intercept	1	15,463.507	162.293	.000	.727	1.00
	Diagnosis	1	2066.291	21.686	.000	.262	.996
	Error	61	95.281				
Percentage on objects							
Within-subject effects	Condition	1	801.001	12.038	.001	.165	.927
	Condition × Diagnosis	1	18.635	.280	.599	.005	.082
	Error	61	66.539				
Between-Subject Effects	Intercept	1	308,032.209	608.601	.000	.909	1.00
	Diagnosis	1	148.333	.293	.590	.005	.083
	Error	61	506.132				
Percentage on bodies							
Within-subject effects	Condition	1	1021.671	30.241	.000	.331	1.00
	Condition × Diagnosis	1	51.142	1.514	.223	.024	.228
	Error	61	33.784				
Between-subject effects	Intercept	1	5650.539	114.091	.000	.652	1.00
	Diagnosis	1	51.412	1.038	.312	.017	.171
	Error	61	49.527				
Percentage on background							
Within-subject effects	Condition	1	49.098	.981	.326	.017	.164
	Condition × Diagnosis	1	29.862	.596	.443	.010	.118
	Error	61	50.067				
Between-subject effects	Intercept	1	121,231.963	558.888	.000	.906	1.00
	Diagnosis	1	799.603	3.686	.672	.060	.471
	Error	61	216.916

Objects (see Table 2 and Fig. 2): We did not detect an effect of diagnosis regarding percentage of fixation on objects (F(1,61) = 0.293; *p* > 0.05). However, we identified a main effect of condition (F(1,61) = 12.038; *p* = 0.001; ηp^2^ = 0.165), where participants looked more at objects during interactive (mean = 52.776, SE = 2.209) compared to parallel condition (mean = 47.655, SE = 2.120). There was no interaction between diagnosis and condition (F(1,61) = 0.280; *p* > 0.05).

Bodies (see Table 2 and Fig. 2): We did not detect an effect of diagnosis on the percentage of fixations on bodies (F(1,61) = 1.038; *p* > 0.05). However, we identified a main effect of condition (F(1, 61) = 30.241; *p* < 0.001; ηp^2^ = 0.331), children looked less at bodies during interactive (mean = 3.909, SE = 0.417) compared to parallel condition (mean = 9.693, SE = 1.091). There was no interaction between diagnosis and condition (F(1,61) = 1.514; *p* > 0.05).

Background (see Table 2 and Fig. 2): We did not identify an effect of diagnosis (F(1,61) = 3.686, *p* > 0.05), condition (F(1,61) = 0.981; *p* > 0.05) or interaction (F(1,61) = 0.596; *p* > 0.05) between diagnosis and condition regarding the time spent looking at the background of the scenes.

### Fixation duration

**Fig. 3:**
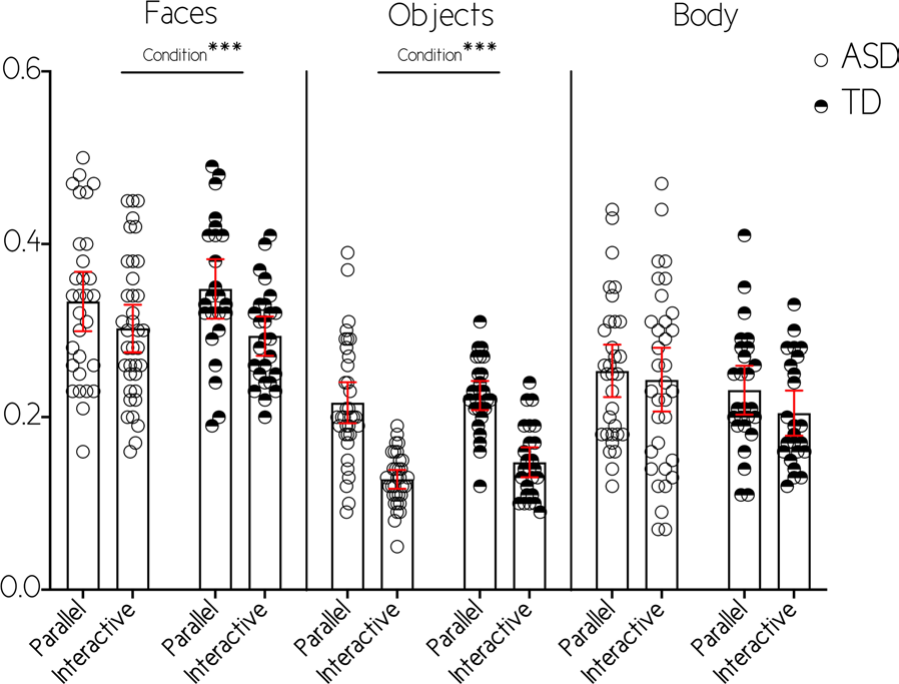
Fixation duration for each AOI in milliseconds. Lines represent variable main effects *p < .05; **p < .01; ***p < .001

Faces (see Table 3 and Fig. 3): We did not identify any effect of diagnosis (F(1, 52) = 0.239; *p* > 0.05). We however identified a main effect of condition (F(1, 52) = 17.766, *p* < 0.001; ηp^2^ = 0.255), fixations were longer during parallel (mean = 0.341, SE = 0.012) compared to interactive condition (mean = 0.287, SE = 0.009). Finally, we did not identify any interaction between condition and diagnosis (F(1, 52) = 0.247; *p* > 0.05).

**Table 3:** Repeated measures linear general model on fixation duration

Measure	Source	*df*	MS	F	*p*	ηp^2^	Observed power
Fixation duration on faces							
Within-subject effects	Condition	1	.77	17.766	.000	.255	.985
	Condition × Diagnosis	1	.001	.247	.621	.005	.078
	Error	52	.004				
Between-subject effects	Intercept	1	10.503	1382.020	.000	.964	1.00
	Diagnosis	1	.002	.239	.627	.005	.077
	Error	52	.008				
Fixation duration on objects							
Within-subject effects	Condition	1	.171	89.993	.000	.621	1.00
	Condition × Diagnosis	1	.000	.082	.775	.001	.059
	Error	55	.002				
Between-subject effects	Intercept	1	3.505	1375.259	.000	.962	1.00
	Diagnosis	1	.009	3.346	.073	.057	.436
	Error	55	.003				
Fixation duration on bodies							
Within-subject effects	Condition	1	.001	.164	.687	.003	.068
	Condition × Diagnosis	1	.000	.019	.890	.000	.052
	Error	53	.007				
Between-subject effects	Intercept	1	5.957	696.078	.000	.929	1.00
	Diagnosis	1	.002	.207	.651	.004	.073
	Error	53	.009

Objects (see Table 3 and Fig. 3): We did not identify any effect of diagnosis (F(1,55) = 0.019; *p* > 0.05). However we identified an effect of condition (F(1,55) = 89.993; *p* < 0.001; ηp^2^ = 0.066), participants having shorter fixation on objects in the interactive condition (mean = 0.138, SE = 0.005) compared to parallel condition (mean = 0.216, SE = 0.007). Finally, there was no interaction between them regarding fixation duration on objects (F(1,55) = 0.000; *p* > 0.05).

Bodies (see Table 3 and Fig. 3): We did not identify an effect of diagnosis (F(1,53) = 0.207; *p* > 0.05), condition (F(1,53) = 0.164; *p* > 0.05) or any interaction (F(1,53) = 0.019; *p* > 0.05) regarding the fixation duration on bodies.

### Number of gaze shifts

**Fig. 4:**
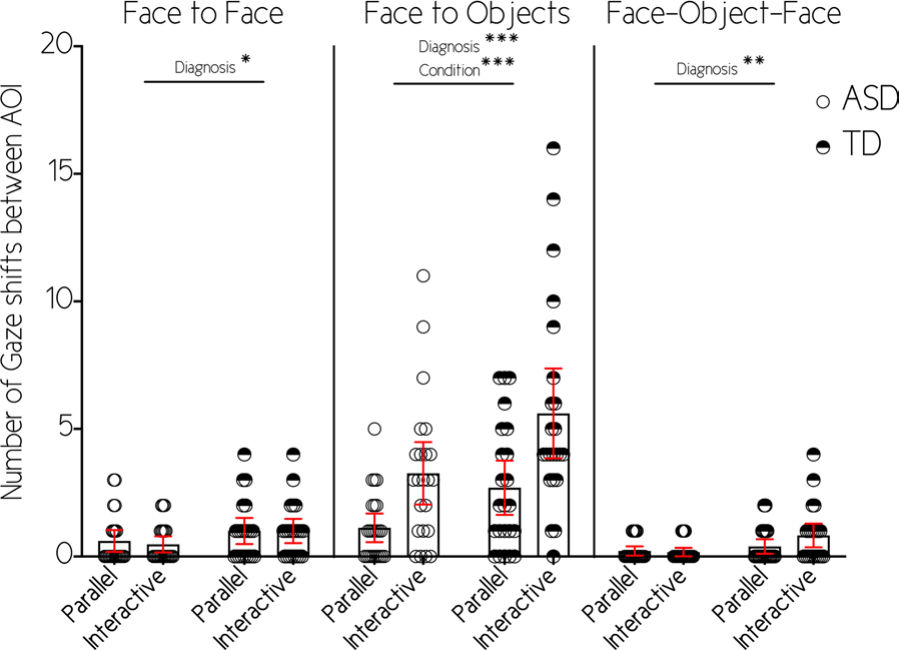
Number of gaze shifts between AOI. Lines represent main effects *p < .05; **p < .01; ***p < .001

F-F shifts (see Fig. 4): We observed an effect of diagnosis (Wald χ^2^(1) = 6.549; *p* = 0.010; *w* = 0.333) where children with ASD shifted less between faces overall compared to TD children. We did not observe a main effect of condition (Wald χ^2^(1) = 0.255; *p* = 0.614), or an interaction between diagnosis and condition (Wald χ^2^(1) = 0.038; *p* = 0.846).

F-O shifts (see Fig. 4): We identified an effect of diagnosis (Wald χ^2^(1) = 13.030; *p* < 0.001; *w* = 0.470), children with ASD shifted less between faces and objects compared to TD children. In addition, we identified a main effect of condition (Wald χ^2^(1) = 29.440; *p* < 0.001; *w* = 0.706), meaning that, in general, children shifted more between faces and objects during interactive compared to parallel condition. However, there was no interaction between diagnosis and condition (Wald χ^2^(1) = 0.993; *p* = 0.319).

F-O-F shifts (see Fig. 4): We identified a main effect of diagnosis (Wald χ^2^(1) = 9.353; *p* = 0.002; *w* = 0.398) where children with ASD made less F-O-F shifts compared to TD children. We did not observe a main effect of condition (Wald χ^2^(1) = 0.474; *p* = 0.491), or an interaction between diagnosis and condition (Wald χ^2^ (1) = 1.520; *p* = 0.218).

### Correlations between viewing patterns and clinical assessments

**Fig. 5:**
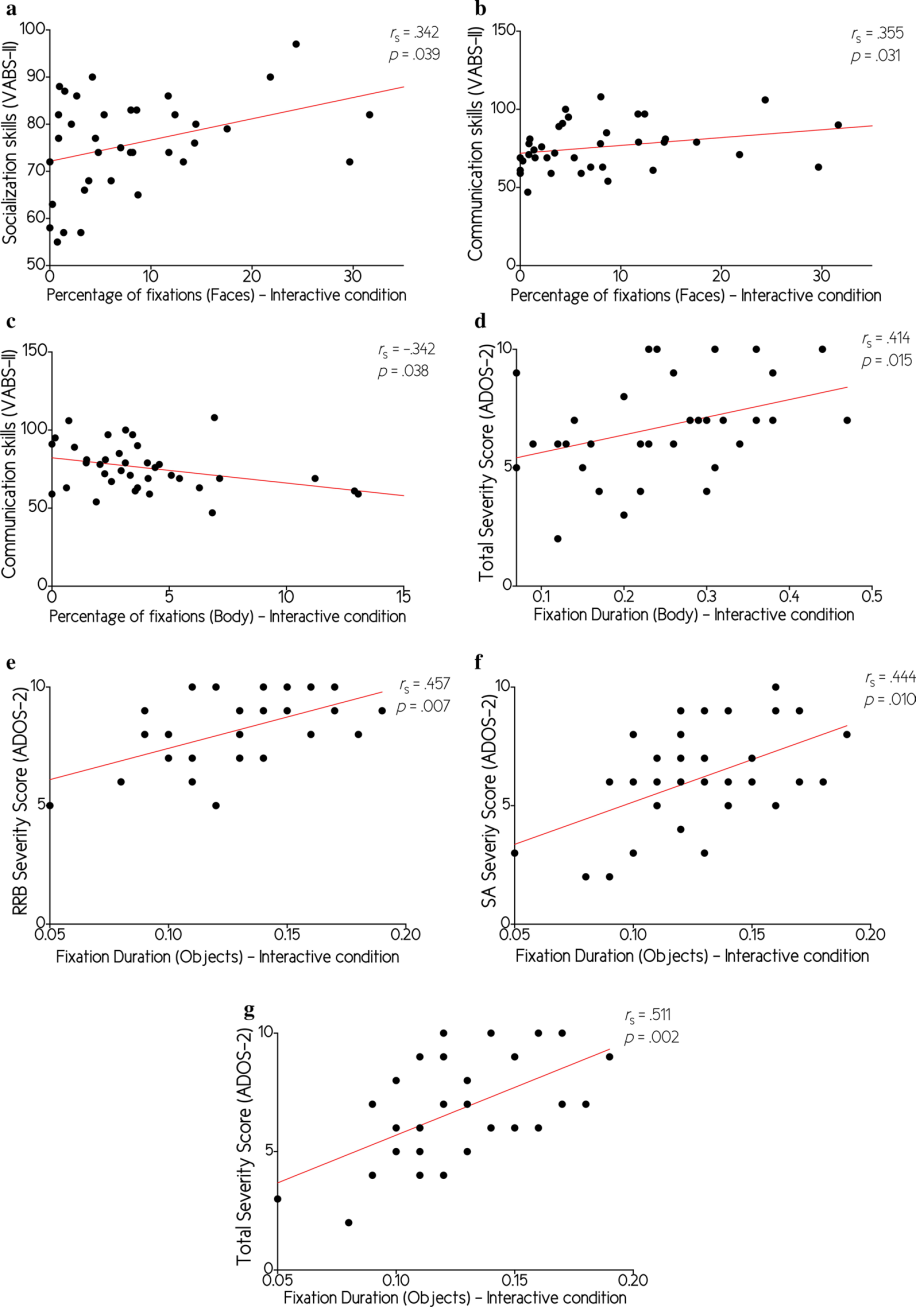
Correlations between eye-tracking and clinical variables. **a** Correlation between Socialization skills (VABS-II) and the percentage of fixations on faces in the Interactive condition; **b** Correlation between Communication skills (VABS-II) and the percentage of fixations on faces in the Interactive condition; **c** Correlation between Communication skills (VABS-II) and the percentage of fixations on bodies in the Interactive condition; **d** Correlation between Total severity score (ADOS-2) and fixation duration on bodies in the Interactive condition; **e** Correlation between Restricted and Repetitive Behavior severity score (ADOS-2) and fixation duration on objects in the Interactive condition; **f** Correlation between Social Affect severity score (ADOS-2) and fixation duration on objects in the Interactive condition; **g** Correlation between Total severity score (ADOS-2) and fixation duration on objects in the Interactive condition

ADOS RRB: We identified a significant correlation between ADOS RRB severity scores and fixation duration on Objects in the interactive condition (*r*_s_ = 0.457; *p* = 0.007, see Table 4 and Fig. 5e) where increased fixation duration was associated with greater RRB symptoms. However, we did not identify any correlation between ADOS RRB severity scores and the fixation percentage on Faces, fixation percentage on Objects, fixation percentage on Bodies, fixation duration on Faces, fixation duration on Objects in the parallel condition, fixation duration on Bodies, F-F shifts, F-O shifts, and F-O-F shifts (all *p* > 0.05, see Table 4).

**Table 4:** Correlations between viewing patterns and clinical assessments

	ADOS			Best Estimate IQ	Vineland-II			
	RRB	SA	Total		Communication	Socialization	Daily living skills	Motor skills
Fixation percentage on faces								
Parallel	− 0.025	− 0.118	− 0.123	− 0.056	0.088	0.138	0.134	0.166
Interactive	− 0.215	− 0.207	− 0.228	0.126	0.355*	0.342*	0.316	0.163
Fixation percentage on objects								
Parallel	− 0.105	− 0.120	− 0.165	− 0.128	0.066	0.004	− 0.060	− 0.143
Interactive	− 0.047	− 0.224	− 0.216	0.061	0.117	− 0.023	0.009	− 0.098
Fixation percentage on bodies								
Parallel	0.183	0.296	0.276	− 0.083	− 0.304	− 0.043	− 0.200	− 0.090
Interactive	0.082	0.308	0.249	− 0.252	− 0.342*	0.008	− 0.117	0.080
Fixation duration on faces								
Parallel	− 0.140	0.257	0.159	− 0.034	− 0.218	0.013	− 0.092	0.142
Interactive	0.001	0.254	0.277	0.060	− 0.101	0.238	0.053	0.164
Fixation duration on objects								
Parallel	− 0.034	− 0.044	0.015	0.213	0.223	0.242	0.161	0.145
Interactive	0.457**	0.444*	0.511**	− 0.061	− 0.126	− 0.151	− 0.233	− 0.197
Fixation duration on bodies								
Parallel	− 0.013	− 0.146	− 0.152	0.281	0.155	− 0.173	0.024	0.004
Interactive	0.232	0.312	0.414*	− 0.089	− 0.171	− 0.099	− 0.112	0.008
Face to Face shift								
Parallel	0.342	− 0.016	0.163	0.254	0.311	0.380	0.208	− 0.002
Interactive	− 0.309	0.004	− 0.081	− 0.128	− 0.168	− 0.031	− 0.202	− 0.168
Face to Objects shift								
Parallel	− 0.113	0.060	0.087	0.396	0.365	0.285	0.294	0.265
Interactive	− 0.088	− 0.043	0.018	0.291	0.316	0.375	0.245	0.170
Face-Object-Face shift								
Parallel	− 0.186	0.057	− 0.049	0.170	0.072	− 0.024	0.016	0.000
Interactive	− 0.203	− 0.133	− 0.141	− 0.173	0.017	0.286	− 0.043	0.026

^*^p < .05, **p < .01

ADOS SA: We identified a significant correlation between ADOS SA severity scores and fixation duration on Objects in the interactive condition (*r*_s_ = 0.444; *p* = 0.010, see Table 4 and Fig. 5f), where increased fixation duration was associated with greater SA symptoms. However, we did not identify any correlation between ADOS SA severity scores and the fixation percentage on Faces, fixation percentage on Objects, fixation percentage on Bodies, fixation duration on Faces, fixation duration on Objects in the parallel condition, fixation duration on Bodies, F-F shifts, F-O shifts, and F-O-F shifts (all *p* > 0.05, see Table 4).

ADOS Total: We identified a significant correlation between ADOS Total severity scores and fixation duration on Objects in the interactive condition (*r*_s_ = 0.511; *p* = 0.002, see Table 4 and Fig. 5g) as well as fixation duration on Bodies in the interactive condition (*r*_s_ = 0.414; *p* = 0.015, see Table 4 and Fig. 5d) where increased fixation duration on were associated with greater symptoms overall. However, we did not identify any correlation between ADOS Total severity scores and the fixation percentage on Faces, fixation percentage on Objects, fixation percentage on Bodies, fixation duration on Faces, fixation duration on Objects in the parallel condition, fixation duration on Bodies in the parallel condition, F-F shifts, F-O shifts, and F-O-F shifts (all *p* > 0.05, see Table 4).

BEIQ: We did not identify any significant correlation between BEIQ and any eye-tracking measure (all *p* > 0.05, see Table 4).

VABS Communication: We identified a significant correlation between VABS Communication scores and fixation percentage on Faces in the interactive condition (*r*_s_ = 0.355; *p* = 0.031, see Table 4 and Fig. 5b) as well as fixation percentage on Bodies in the interactive condition (*r*_s_ = − 0.342; *p* = 0.038, see Table 4 and Fig. 5c) where increased fixation percentage on faces where associated with greater communication skills whereas increased fixation percentage on bodies where associated with lower communication skills. However, we did not identify any correlation between VABS Communication scores and the fixation percentage on Faces in the parallel condition, fixation percentage on Objects, fixation percentage on Bodies in the parallel condition, fixation duration on Faces, fixation duration on Objects, fixation duration on Bodies Faces, F-F shifts, F-O shifts, and F-O-F shifts (all *p* > 0.05, see Table 4).

VABS Socialization: We identified a significant correlation between VABS Socialization scores and fixation percentage on Faces in the interactive condition (*r*_s_ = 0.342; *p* = 0.039, see Table 4 and Fig. 5a) where increased fixation percentage on faces where associated with greater socialization skills. However, we did not identify any correlation between VABS Socialization scores and the fixation percentage on Faces in the parallel condition, fixation percentage on Objects, fixation percentage on Bodies, fixation duration on Faces, fixation duration on Objects, fixation duration on Bodies, F-F shifts, F-O shifts, and F-O-F shifts (all *p* > 0.05, see Table 4).

VABS Daily living skills: We did not identify any significant correlation between VABS Daily Living Skills scores and any eye-tracking measure (all *p* > 0.05, see Table 4).

VABS Motor skills: We did not identify any significant correlation between VABS Motor skills scores and any eye-tracking measure (all *p* > 0.05, see Table 4).

## Discussion

Our results demonstrate decreased social orienting towards faces in ASD compared to TD, which is commensurate with previous studies [2–9, 34, 52]. Similar to Harrop et al.’s results [27], our preschool boys with ASD showed reduced attention to faces compared to TD children. Watching a social interaction tended to increase time spent on faces in both groups in Harrop et al.’s study, an effect that we did not observe in our sample. Apart from a slight non-significant increase, watching a social interaction had no impact on the time spent looking at faces. A background difference used in the two studies may explain these contradictory results. While Harrop et al. [27] used a rich background that included various objects, the background we used was very neutral and did not include objects. A rich background could bias attention towards non-socially relevant elements, especially in individuals with ASD who present disrupted low-level perception, which can enhance spatial contrast sensitivity (for a review see [53]). Accordingly, the neutral background used in our study may have reduced potential distractions, leading to increased focus on the social elements of the scene even during the parallel condition. Furthermore, similar to previous studies (e.g. [22, 34]), we observed correlations between socialization and communication skills and percentage of fixations on faces exclusively during the interactive condition. Preschoolers who were attending more to faces also had stronger social and communication skills. These results reinforce the idea that ASD children’s visual exploration of their social environment impacts the development of their socio-communicative skills. Moreover, this suggests that only during a socially complex task is the relationship between visual exploration patterns and socio-communicative deficits in children with ASD visible. Taken together, our results support interventions that are based on structured and neutral teaching environments,such as Treatment and Education of Autistic and Communication-Handicapped Children for example (TEACCH) [54] which reduce environmental distractions and maintain a child’s attention on the task at hand,but also reinforce the idea that social interaction tasks are most relevant in appraising social deficits in ASD [2].

We did not observe group differences pertaining to the percentage of time spent looking at objects, bodies or background. However, we observed a similar tendency in both groups to reduce the time spent on bodies in favor of time spent on objects when watching an interaction compared to parallel play. This attentional shift, from bodies to objects, represents a direct consequence of increased social complexity and shared attention of the actors in the scene. Therefore, it was interesting to observe that, in our sample, shared attention of the actors during interactive play caused a similar visual focus on objects in both groups, whereas children with ASD showed decreased time spent looking at the shared activity in Shic et al.’s study [16]. There are several potential explanations for this discrepancy. The first, as previously discussed, might be related to the more visually complicated background in Shic et al.’s study, as children with ASD spent increased time looking at the more complicated background, consequently compromising the time spent looking at the activity. Second, an alternate explanation may relate to the fact that in Shic et al.’s study, the actors involved in the task (an adult and a child) interacted vocally, with the adult encouraging the child to solve a puzzle. By contrast, in our interactive condition, the children in the videos interacted non-verbally only by looking at each other. Moreover, unlike the puzzle in Shic’s study, which is a silent game, our activity was playing a xylophone therefore producing musical tone. Considering that presenting a congruent sound induces faster orientation towards a target stimulus during a visual exploration task [55] and that children with ASD demonstrate atypical auditory processing with preserved or heightened abilities in musical processing [56, 57], the auditory component might have biased attentional focus in the two paradigms in different ways. Third, Shic et al. [16] proposed that children attend to elements that are within reach of their ability to comprehend, according to McCall and McGhee's [58] moderate discrepancy hypothesis, which could result in reduced attention to the shared activity especially in children with higher symptom severity. In contrast to Shic’s task, the activity presented in our study might not have induced such a bias, given its simplicity. On a related note, we observed a negative correlation between communication skills and percentage of fixations on bodies, exclusively during the interactive condition. This suggests that the children who were less sensitive to context modulation and shared attention, who kept watching non-socially relevant elements of the scene, had decreased communication abilities. Taken together, our results suggest that ASD and TD’s visual exploration patterns were both affected by social complexity but that a decreased sensitivity to this context modulation might impact the development of communication skills.

In addition to modify time spent on AOI, social complexity also impacted visual exploration patterns’ dynamism. Indeed, despite a similar time spent on faces, social complexity impacted the fixation duration on faces in our sample where we observed a decrease of fixation duration in the interactive condition compared to the parallel one. In addition, children in our sample also decreased their fixation duration on objects during the interactive condition despite an increase of time spent on objects. On the other hand, while children decreased their time spent on bodies in the interactive condition, we did not observe any diminution of fixation duration on bodies. These results suggest that social complexity had a strong effect on the dynamic visual exploration on socially relevant AOI such as faces and objects but that it did not modify the visual exploration dynamism regarding non-socially relevant AOI such as bodies. Taken together, these results suggest that increased social complexity involves a modification of attentional engagement on social areas as reflected by the reduction of fixation duration. In our sample, children who displayed longer fixation on non-socially relevant AOI such as body had higher level of symptoms. Similarly, longer fixations on objects in the interactive condition were associated with a higher level of symptoms as well. Considering that several studies shown that children with ASD display visual disengagement difficulties [37], our results might suggest that attentional difficulties may well constitute a core symptom of ASD as children exhibiting more symptoms appear to be less sensitive to social complexity attentional modulation.

Per our hypothesis, decreased duration of fixations should have impacted participants’ flexibility to become more active in their exploration between socially relevant AOI, consequently increasing the gaze shifts between faces as well as between faces and objects. However, although we only selected children with ASD with a minimal interest for faces, we still observed diminished social monitoring in our ASD group compared to our TD group, measured by the number of spontaneous Face to Face (F-F) shifts. This suggests that despite a minimal social orientation, children with ASD display an atypical dynamic exploration of social scenes. Conversely, we observed a similar number of F-F shifts regardless of the social context, suggesting that social monitoring is not modulated by context but is, rather, intrinsic in typical development. Considering the aforementioned positive relationship between socio-communicative skills and percentage of fixations on faces during interaction, this early decreased social monitoring observed in ASD might represent a basic alteration linked to impaired development of many higher social skills in ASD, such as shared intentionality [59, 60] or social oddity detection [61]. Given that most of these “higher” skills rely on the dynamic exploration of social interactions to identify relevant social cues, a decreased shift between faces, where most non-verbal communication occurs, is likely to have a detrimental effect on these skills.

While social complexity did not impact the number of F-F shift, we observed that children increased their Face to Objects (F-O) shifts, coherent with action monitoring and joint attention behaviors expected during an interactive play. Per our hypothesis, actors’ shared attention on a common object in the interactive condition, increased its social saliency and increased the number of joint attention behaviors. However, joint attention behaviors were still less frequent in the ASD group compared to the TD group, as reported in most studies on joint attention and ASD [19, 21–24, 62]. An overall decrease in F-O gaze shifts supports joint attention deficits as a core feature of ASD. Nevertheless, it was encouraging to see that, despite these fundamental difficulties, social complexity still elicited a slight increase in joint attention behaviors in the ASD group. This supports some preserved joint attention abilities in ASD that are more apparent during the viewing of a richer social scene. Considering this, our results support studies and therapies that advocate for the inclusion of exaggerated emotional expressions and very rich social interactions during social interventions designed for children with ASD (e.g. Early Start Denver Model) [63] as they might recruit more of the children’s skills.

Finally, we observed significantly less Face to Objects to Face (F-O-F) shifts, or three-step joint attention gaze shifting in our ASD group compared to our TD group. This three-step joint attention shifting reflects a greater level of complexity involving higher socio-cognitive skills. Considering that joint attention and social cognition are developmental processes [18, 21, 64] and that oculomotor motricity depends on brain regions that develop during infancy [65, 66], it is very likely that three-steps joint attention shifts develop later after less sophisticated shifts have been mastered (e.g., F-O shifts). Therefore, the decreased occurrence of lower-level shifts observed in our ASD sample may explain why we observed few F-O-F shifts but also why social complexity did not increase these shifts overall. If we had included subjects with a wider age range in our ASD group, older children who demonstrate a TD-like frequency of two-step gaze shifts, we may have observed a more complex exploration of others' actions, including three-step gaze shifts that are modulated by context. Including older subjects as well could lead to a deeper understanding of the link between dynamic exploration patterns and the development of high-level social behaviors. Taken together, our results support joint attention as a pivotal ability in the development of higher socio-communicative skills during childhood and its importance as a key target in early interventions [67],for a review see [63, 68].

### Limitations

The main limitation of this study concerns its small sample size, which does not allow statistically robust conclusions to be drawn. Considering the small sample size, results should be taken with caution. Post-hoc power analyses were performed using the software package, G*Power3 [69] using the present sample size of 63, with 2 groups at an α of 0.05. The recommended effect sizes used for this assessment were as follows: small (f = 0.10), medium (f = 0.25), and large (f = 0.40) [70]. The post hoc analyses revealed the statistical power for this study was 0.35 for detecting a small effect, 0.97 for detecting a medium effect and 0.99 for detecting a large effect size. In consequence, there was adequate power (> 0.80) at the medium and large effect size level but not enough statistical power to detect small effect sizes. Additional power analysis using similar parameters showed that in order to reach a power of 0.80 for small effect sizes, sample size should be increased to 200 participants.

Another limitation is the exclusion of females from our sample. Considering previous results showing that males with ASD consistently exhibit decreased attention to faces while females with ASD show TD-like attendance to faces when watching parallel play [27], it is important to keep in mind that our results apply only to males and can not be extended to females with ASD.

In our study, we quantified the number of gaze shifts and interpreted them as spontaneous joint attention behaviors or attempts at grasping non-verbal communication cues. Another way to analyze these gaze shifts could be to investigate whether they occur in coordination with non-verbal communication cues. This could provide information about whether higher levels of gaze shifting contingency with communication cues are related to better socio-communicative skills in ASD. However, this was not feasible in our study because there was no communication at all during the parallel play condition, making the analyses irrelevant. A possible workaround for future studies focusing on context modulation who would like to investigate this could be to manipulate levels of non-verbal communication behaviors between actors among conditions.

In spite of our efforts to control many aspects; including two children of the same age, a boy and a girl who look alike because they are twins, not changing the game proposed in both conditions, keeping the same furniture and the same room; the scenes differ in some aspects, such as the distance and the position of the children in the room. It is therefore not possible to exclude the fact that some of the results discussed in this article might be related to these changes.

Finally, we agree with the observation proposed by Parish-Morris et al. [29] who pointed out that screen-based eye-tracking studies are still lacking some ecological validity. Despite our efforts to propose paradigms as close as possible to everyday life, it is difficult to know whether the visual exploration patterns described in our study reflect authentic visual exploration of others' actions in a real-life situation. To remedy this, future studies could try live interactions using experimenters, or take advantage of new wearable eye-tracking devices, although feasibility remains questionable when applied to toddlers or preschoolers with ASD.

## Conclusion

This study uses a naturalistic design to study the visual exploration of others' actions, the primary source of social learning during early development. We manipulated context by presenting a socially simple scene of two children doing parallel play and a more complex social scene of the two children doing interactive play. We observed reduced attention to faces in the ASD group associated with decreased socio-communicative skills, and atypical dynamic exploration of others' actions as they exhibited less spontaneous gaze shifts suggesting reduced attention to non-verbal communication cues and lower joint attention skills. In addition, children who were less sensitive to social complexity attentional modulation showed longer fixations associated with higher level of ASD symptoms. The examination of spontaneous gaze shifts in a naturalistic design can help future understanding of how children with ASD dynamically process interactions to guide future interventions. This study supports interventions targeting the development of joint attention skills and the inclusion of engaging social interactions to reduce social deficits in ASD given the positive effects on visual exploration patterns of children with ASD of viewing interactive play compared to parallel play.

## Data Availability

The datasets analyzed during the current study includes sensitive data collected in a local sample. The risk of breaching patient confidentiality would be high; therefore, datasets are not publicly available but are available from the corresponding author on reasonable request.

## Abbreviations

ADHD: Attention deficit-hyperactivity disorder
ADOS: Autism diagnostic observation schedule
AOI: Area of interest
ASD: Autism spectrum disorder
BEIQ: Best estimate intellectual quotient
F-F: Face to Face gaze shifts
F-O: Face to Objects gaze shifts
F-O-F: Face to Objects to Face gaze shifts
MSEL: Mullen Scales of Early Learning
PEP-3: Psycho-Educational Profile, third edition
RRB: Restricted and repetitive behavior
SA: Social affect
SD: Standard deviation
TD: Typical development
VABS-II: Vineland Adaptive Behavior Scales, second edition
WPPSI-IV: Wechsler Preschool and Primary Scale of Intelligence, fourth edition
